# Complete Genome Sequence of *Klebsiella pneumoniae* Strain HKUOPLC, a Cellulose-Degrading Bacterium Isolated from Giant Panda Feces

**DOI:** 10.1128/genomeA.01318-15

**Published:** 2015-11-12

**Authors:** Matthew Guan-Xi Lu, Jingwei Jiang, Lirui Liu, Angel Po-Yee Ma, Frederick Chi-Ching Leung

**Affiliations:** a The Independent Schools Foundation Academy, Hong Kong SAR, China; b School of Biological Sciences, University of Hong Kong, Hong Kong SAR, China; c Bioinformatics Centre, Nanjing Agricultural University, Nanjing, China

## Abstract

We report here the complete genome sequence of *Klebsiella pneumoniae* strain HKUOPLC, isolated from a giant panda fecal sample collected from Ocean Park, Hong Kong. The complete genome of this bacterium may contribute to the discovery of efficient cellulose-degrading pathways.

## GENOME ANNOUNCEMENT

Strain HKUOPLC was originally determined by a functional assay involving a decolorizing ring in the identification medium with CMC-Congo red and then screening based on its ability to grow in minimal basal-salt medium with Whatman filter paper grade 1 supplied as the sole carbon source under aerobic conditions (1). It was identified as *Klebsiella pneumoniae* by comparing its sequence to the nucleotide database in NCBI using BLASTn; the BLAST result showed the highest identity, at 95%, to strain ATCC 43816 KPPR1 (NCBI accession no. CP009208) (2). The genus *Klebsiella* is facultative anaerobic, having both respiratory and fermentative types of metabolism. Most strains produce acid, gas, or 2,3-butanediol as a major end product of glucose fermentation (3).

The genomic DNA of strain HKUOPLC was extracted using the cetyltrimethylammonium bromide (CTAB) protocol (4) from a pure aerobic culture in Luria broth. Its quality and quantity were examined and measured using a NanoDrop2000 spectrophotometer (Thermo Scientific) and the Quant-iT PicoGreen double-stranded DNA (dsDNA) kit (Invitrogen), respectively. A 2 × 300 MiSeq library and a MiSeq 8-kb paired-end library were constructed and sequenced with the Illumina MiSeq platform at the Bioinformatics Centre, Nanjing Agricultural University, Nanjing, China. The Illumina MiSeq platform achieved 768,637 reads that were >250 bp, with a mean quality score >30. These reads were assembled using the Newbler version 2.7 assembly software program (Roche) into 20 large contigs with 40-fold coverage of 5,056,441 bases. Gaps between the contigs were closed by bioinformatics tools and subsequently assembled using the SeqMan software (DNAStar).

The complete genome of strain HKUOPLC was submitted to the NCBI Prokaryotic Genome Automatic Annotation Pipeline (PGAAP) for annotation. *K. pneumoniae* strain HKUOPLC has one chromosome, which is 5,088,873 bp in size, with a G+C content of 58.04%. The genome has a total of 4,962 genes, 4,764 predicted coding sequences (CDSs), 92 pseudogenes, and 43 frameshifted genes. Some other features were identified, including 79 tRNAs, 24 rRNAs, and 3 noncoding RNAs (ncRNAs).

### Nucleotide sequence accession number.

The complete genome sequence of *K. pneumoniae* strain HKUOPLC has been deposited in GenBank under the accession no. CP012300 for the chromosome. The version described in this study is the first version.
